# Oral efficacy of controlled-release doxycycline against *Ichthyophthirius multifiliis* infestation in salmonids

**DOI:** 10.17221/104/2025-VETMED

**Published:** 2026-04-27

**Authors:** Zuzana Mikulkova, Katerina Matejickova, Jitka Motlova, Pavlina Ginterova, Martin Jerabek, Lubomir Pojezdal

**Affiliations:** ^1^Tekro, spol. s r.o., Prague, Czech Republic; ^2^Department of Infectious Diseases and Preventive Medicine, Veterinary Research Institute, Brno, Czech Republic

**Keywords:** brook trout, controlled release drug, medicated diet, rainbow trout

## Abstract

Ichthyophthiriosis, caused by *Ichthyophthirius multifiliis*, is a major threat in salmonid aquaculture. This study evaluated the efficacy of orally administered standard doxycycline and controlled-release doxycycline formulations in experimentally infected brook trout (*Salvelinus fontinalis*). Fish received medicated feed for 10 days starting seven days post-infection. Controlled-release doxycycline achieved the highest survival rate (84.2%), followed by standard doxycycline (73.7%), while no control fish survived after day 25 post-infection. Both drug formulations eliminated trophonts from the skin and gills by 20 days post-treatment. Despite a 2.5-fold lower dietary dose, the controlled-release doxycycline achieved a tissue concentration equivalent to ~32% of that of standard doxycycline, with similar retention rates. Levels of doxycycline residues in water declined over time for both groups, indicating limited environmental release. These results demonstrate the effective mitigation of ichthyophthiriosis with orally administered doxycycline and the enhancement of antiparasitic efficacy and reduction of drug load in tissues and the environment via controlled-release technology, supporting its potential as a sustainable medicated feed strategy in salmonid aquaculture.

Salmonids, such as rainbow (*Oncorhynchus mykiss*) and brook trout (*Salvelinus fontinalis*), are key freshwater species in European aquaculture due to their rapid growth, reliable reproduction, and high meat quality (Hough 2022). With increasing intensification of farming systems, disease outbreaks leading to substantial economic losses have become more frequent (Buchmann 2022). Among the most problematic pathogens is the ciliate *Ichthyophthirius multifiliis* (*Ich*)*,* which causes ichthyophthiriosis (white spot disease) and is particularly harmful to juvenile fish, often resulting in high mortality or complete cohort collapse (Abdel-Hafez et al. 2014).

Malachite green, once an effective treatment against *Ich*, has been banned for all food-fish life stages due to its carcinogenic and mutagenic properties (European Commission 2023), necessitating alternative control options. Deployment of chemotherapeutics could represent a potential approach, with the antibiotic doxycycline shown to significantly reduce mortality in infected rainbow trout (Abdel-Hafez et al. 2014). Although antimicrobials can be administered via various routes, feed-based delivery is often preferred due to reduced stress and total dosage, compared to bath treatment regimes. Nevertheless, peroral application can be limited by incomplete digestion and environmental release (Ibrahim et al. 2020). The utilisation of controlled-release formulations has the potential to mitigate these aspects, offering a more sustainable approach to parasite control in aquaculture (Neto et al. 2019).

The present study, therefore, aimed to assess the efficacy of doxycycline against ichthyophthiriosis when delivered orally using microgranulated feed with controlled-release technology.

## MATERIAL AND METHODS

### Source of parasite

Juvenile rainbow trout (*Oncorhynchus mykiss*) naturally infected with *Ichthyophthirius multifiliis* and free of other ectoparasites were obtained from a commercial fish farm and used as the source of infection. The *Ich* population was maintained under controlled laboratory conditions by serial passage on naïve brook trout (*Salvelinus fontinalis*).

### Experimental infection design

**Figure 1:**
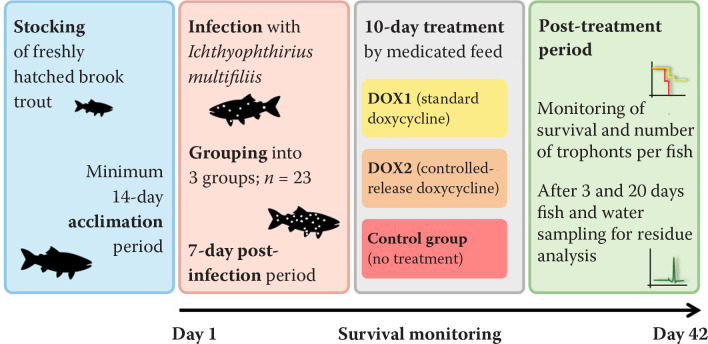
Overview of the experimental timeline

Brook trout fry were obtained from a commercial hatchery, acclimated for at least 14 days, and screened for ectoparasites with negative results. The experimental infection study was conducted to evaluate the efficacy of medicated feed containing standard doxycycline (DOX1) compared with feed containing controlled-release doxycycline (DOX2). On the day of infection, 69 healthy brook trout (body length 7–9 cm, mean body weight 6.0 g) were exposed to *Ich* by 24-hour cohabitation with infected brook trout. Afterwards, fish were randomly distributed into three groups (*n* = 23) in 80 l glass aquaria with dechlorinated water, with a total system volume (including filtration and tubing) of approximately 96 l. All fish were kept at 13 °C under a 12 h light/12 h dark cycle for 42 days. Fish were fed commercial pellet feed (Inicio plus M, 0.8 mm; Biomar Group, Aarhus, Denmark) at 2% of body weight once daily. At seven DPI (days post-infection, in the preclinical phase), fish were fed medicated feeds (DOX1, DOX2, or Control) once daily at 2% of body weight for 10 consecutive days, with feed intake monitored visually. The Control group was fed the same commercial feed without medication throughout the experiment (Figure 1).

### Preparation and administration of medicated feed

Medicated feed was manually prepared by adding an analytical standard of doxycycline hyclate (DOX1, purity >95%; Merck, Darmstadt, Germany) to commercial pellets (Inicio plus M, 0.8 mm; Biomar Group, Aarhus, Denmark) at a dose of 2.0 mg/g of feed. Due to the low water solubility of doxycycline, DOX1 was incorporated into the pellets using rapeseed oil and thoroughly vortex-mixed at 3 200 rpm for 5 minutes. The second medicated feed was prepared using the same procedure but contained doxycycline hyclate in a controlled-release formulation (DOX2, 40% w/w active substance content, particle size of up to 0.2 mm). The controlled-release matrix was produced using high-shear mixing followed by fluidised-bed processing, in which the active substance was combined with a 4 : 6 mixture of fatty acids and anhydrous dicalcium phosphate. The final concentration of DOX2 in the feed was 0.8 mg/g.

### Sample collection

Water temperature, oxygen saturation, and pH were recorded daily in each aquarium. Dead fish were removed daily, and mucus samples were collected from each carcass by scraping one lateral side of the fish for microscopic examination (40× magnification) to confirm the presence of *Ich* trophonts. At three time points – prior to treatment, three days post-treatment, and 20 days post-treatment, two fish per group were randomly selected, euthanised with an overdose of benzocaine, and examined microscopically for *Ich* trophont counts. Carcasses were stored at –80 °C for pharmacological analysis. At two time points, three and 20 days post-treatment, 40 ml of water was collected from each aquarium and stored at –80 °C for DOX content analysis. All surviving fish were euthanised by benzocaine overdose.

### Ethical approval

All experiments were approved by the Branch Commission for Animal Welfare of the Ministry of Agriculture of the Czech Republic, Permission No. Mze 2407.

### Residue analysis

Concentrations of DOX1 and DOX2 in whole-fish homogenate, dissected muscle with skin, and water were determined by LC-MS/MS using an Agilent 1290 Infinity LC system coupled with an Agilent 6460 TripleQuad LC/MS. Chromatographic separation was achieved on a Luna Omega C18 column (1.6 μm; 2.1 × 100 mm) at 40 °C, using a mobile phase of 0.1% formic acid in water and 0.1% formic acid in methanol at a flow rate of 0.35 ml/min. Doxycycline concentrations were quantified using Agilent MassHunter Workstation (quantitative analysis).

Sample preparation followed a modified QuEChERS protocol based on Kim et al. (2021). Briefly, homogenised tissue (0.5 g) was extracted with acidified acetonitrile in the presence of inorganic salts. Methacycline (approx. 10 ppm) was used as an internal standard.

After phase separation, an aliquot of the acetonitrile layer was used for LC-MS/MS analysis. Water samples were thawed, filtered (0.45 μm), and analysed directly.

Mean concentrations were calculated from duplicate injections. The method was used to assess the persistence of doxycycline residues in fish tissues and water at 3 and 20 days post-treatment. The limit of quantification (LOQ) was <50 μg/kg for whole-fish homogenate and muscle with skin, and <2 μg/l for water samples.

### Statistical analysis

Survival functions were estimated using the Kaplan–Meier method. Differences in survival between groups were assessed with the pairwise Mantel–Haenszel log-rank test. All statistical analyses were performed using the survival and *survminer* packages in R (v4.5.1; R Project for Statistical Computing, Vienna, Austria). Kaplan–Meier survival curves were generated using the *ggplot2* package in R (v4.5.1). A significance level of α = 0.05 was considered for all statistical tests.

Mortality prediction was assessed using a *Z*-statistic, calculated as *Z* = (*O* − *E*)/√var(*O* − *E*), where *O* represents the observed number of mortalities, *E* the expected number, and var(*O* − *E*) the variance of the difference between observed and expected values.

## RESULTS

**Figure 2:**
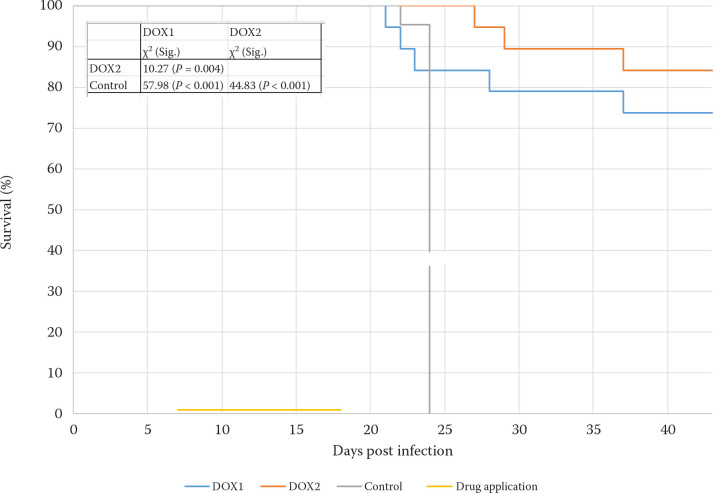
Kaplan–Meier survival curves of brook trout infected with *Ichthyophthirius multifiliis*

The controlled-release doxycycline group (DOX2) exhibited the highest total survival rate (84.2%) throughout the observation period, followed by the standard doxycycline group (DOX1) with 73.7%. The Control group showed a dramatic decline in survival, reaching a median survival of only 24 days and a final survival rate of 0% (Table 1, Figure 2). The Kaplan–Meier curve analysis confirmed a highly significant survival benefit in both DOX-treated groups compared with the Control (*P* < 0.001), supported by negative *Z*-statistics in mortality prediction, indicating a strong protective effect of doxycycline treatment. A statistically significant difference (*P* = 0.004) was also observed between the DOX1 and DOX2 groups (Figure 2), suggesting that the controlled-release formulation of doxycycline (DOX2) provided superior protection against *Ich* infection, despite containing 2.5 times lower doxycycline concentrations than the standard formulation (DOX1).

**Table 1:** Effect of medicated feed on the mortality of brook trout infested with Ichthyophthirius multifiliis

Group	Diet (dose)	Total survival (%)	Median of survival	Significance *P*-value log-rank test	Mortality prediction *Z*-stat
DOX1	doxycycline (2 mg/g feed)	73.7	>42 days	Sig. (*P* < 0.001)	–5.78
DOX2	doxycycline controlled release (0.8 mg/g feed)	84.2	>42 days	Sig. (*P* < 0.001)	–8.70
Control	–	0	24 days	–	14.47

Statistical significance was assessed using Kaplan–Meier survival analysis, comparing the Control group with the other groups; Mortality prediction: Negative *Z*-stat values indicate a survival advantage relative to the Control group, whereas positive values indicate higher mortality risk

On day 7 post-infection, fish were treated with a medicated diet containing either standard doxycycline (DOX1) or controlled-release doxycycline (DOX2) for 10 days. Control fish were fed the same commercial pellet diet without medication. Differences between Kaplan–Meier curves were evaluated for statistical significance using the Mantel–Haenszel log-rank test, with test statistics shown in the upper left panel of the figure.

Prior to treatment (day 5 post-infection), the presence of *Ich* trophonts was confirmed by microscopic examination of two fish, which showed 25 and 35 trophonts per mucus smear from one lateral side of each fish. Medicated feeding with both doxycycline formulations had a marked effect on the mean number of trophonts present on skin and gills per fish during the post-treatment period (Table 2). As early as three days post-treatment, a reduction in trophont counts on the gills per fish was observed, especially in the DOX2 group. Twenty days post-treatment, dead fish in the DOX2 group exhibited low numbers of trophonts on skin and gills, while parasitological examination of surviving fish revealed no detectable trophonts on the skin or gills in either DOX-treated group. This absence persisted even 26 days post-treatment, confirming the long-lasting protective effect of both doxycycline formulations against *Ich* infection.

**Table 2:** Effect of medicated feeds on the survival rate and number of trophonts per brook trout infested with *Ichthyophthirius multifiliis* during the post-treatment period

Days post-treatment (day post-infection)	Survival (%)				Number of trophonts per fish		
	DOX1	DOX2	Control		DOX1	DOX2	Control
3 (19)	100	100	100		S: 155, G: 4 S: 103, G: 6	S: 80, G: 5 S: 25, G: 2	S: 51, G: 22 S: 204, G: 52
8 (24)	84.2	100	0		–	–	S: 22 ± 24 G: 22 ± 16
12 (28)	79.0	94.7	0		–	S: 22, G: 6 (*)	–
20 (36)	79.0	89.5	0		S: 0, G: 0 S: 0, G: 0 S: 0, G: 0 (*)	S: 0, G: 0 S: 0, G: 0 S: 6, G: 7 (*)	–,– –,–
26 (42)	73.7	84.2	0		S: 0, G: 0 S: 0, G: 0	S: 0, G: 0 S: 0, G: 0	–,– –,–

Fish were treated with standard doxycycline (DOX1) or controlled-release doxycycline (DOX2); Control fish received the same commercial diet without medication

(*) = dead fish; G = gill; S = skin

Concentrations of doxycycline in fish tissues, whole fish homogenates, and water samples after feeding with standard (DOX1) and the controlled-release formulation (DOX2) are summarised in Table 3. Three days post-treatment, measurable concentrations of both doxycycline forms were detected in all matrices. The highest concentrations were observed in whole-fish homogenates, followed by muscle, skin, and water. The concentration of doxycycline in whole-fish homogenates was approximately threefold higher than in muscle with skin for both formulations. Despite being administered at a 2.5-fold lower dietary concentration (0.8 mg/g vs 2.0 mg/g feed), the controlled-release formulation DOX2 achieved tissue concentrations reaching approximately 32% of those measured in the DOX1 group at 3 days post-treatment. Based on the feeding rate (2% of body weight), a mean body weight of 6 g, and a 10-day administration period, the total estimated doxycycline intake per fish was 2.4 mg for DOX1 and 0.96 mg for DOX2. Using the mean measured concentrations in whole fish homogenates, the retained amount of doxycycline in the fish body was calculated relative to the administered dose, representing approximately 0.12% for DOX1 and 0.10% for DOX2. Doxycycline concentrations in water were comparable between both treatment groups at day 3 (70.0 μg/l for DOX1 and 80.7 μg/l for DOX2).

**Table 3:** Concentrations of standard doxycycline (DOX1) and controlled-release doxycycline (DOX2) in tissues, whole fish homogenate, and water samples of brook trout during the post-treatment period

Day post-treatment	Analyte	Fish	Concentration (μg/kg) in the muscle with skin	Concentration (μg/kg) in whole fish	Concentration (μg/l) in water
3	DOX1 2.0 mg/g feed	1	188.0	644.5	70.0
		2	73.9	308.1	
20		1	<50.0	<50.0	30.5
		2	<50.0	<50.0	
					
3	DOX2 0.8 mg/g feed	1	51.7	172.8	80.7
		2	51.1	135.9	
20		1	<50.0	<50.0	18.6
		2	<50.0	<50.0	

<50.0 = values under the limit of detection

At 20 days post-treatment, doxycycline concentrations in all fish samples dropped below the quantification limit (<50 μg/kg), while low residual levels persisted in water (30.5 μg/l in the DOX1 tank and 18.6 μg/l in the DOX2 tank), indicating limited but prolonged environmental release. Between 3 and 20 days post-treatment, water concentrations decreased by 56% in DOX1 and by 77% in DOX2.

## DISCUSSION

This work examines the efficacy of orally administered doxycycline in standard and controlled-release applications during infection trials by evaluating the ichthyophthiriasis survival rates, trophont counts and DOX residues in various matrices.

Although the efficacy of doxycycline against the *Ich* infestation observed in this study was also confirmed by Abdel-Hafez et al. (2014), the specific mechanism of action against the parasite is yet to be described. The effect of DOX is well characterised in the context of antimalarial therapy (*Plasmodium* spp.) in humans, where the drug inhibits the protein synthesis inside the apicoplast containing a prokaryotic-like 70S ribosomal unit, thus impairing the critical metabolic pathways and ultimately interfering with the protozoans life cycle (Okada et al. 2020). Despite the obvious absence of the apicoplast organelle in ciliates, a similar effect targeting the ribosomes might play a role in the case of *I. multifiliis*, especially due to the structural similarity of the *Ich* and bacterial ribosomes. Antimicrobial properties of DOX also possess the ability to interfere with the proposed endosymbiotic rickettsial bacteria observed within trophonts and theronts and the possibly pathogenic or opportunistic bacteria found on the surface of the parasite or in the environment (Sun et al. 2009). Lastly, tetracycline antibiotics have been shown to modulate the immune response in salmonids, with this effect potentially positively contributing to the resolution of the *Ich* infestation (Lunden et al. 1998).

In Europe, no drug containing DOX is currently registered for application in aquaculture. Any use of the antimicrobial is therefore considered off-label and needs to comply with the established minimal residue limits and withdrawal periods for the target species (European Commission 2015). Additionally, DOX’s ability to persist in the aquatic environment and affect microbial populations even at low residual concentrations, along with the general problem of increasing antimicrobial resistance among pathogens worldwide, calls for a judicious use of the drug in aquaculture. High-value individuals, such as broodstock, and cases with clearly diagnosed causative pathogens with analysed antimicrobial resistance profiles should be preferred targets for the treatment (FAO 2019).

After the ban of malachite green in European aquaculture (EU 2023/411), the possibilities of ichthyophthiriasis treatment in farmed fish became severely limited. Although effective, the zoohygienic measures, such as lower stock densities, increased water flow-through, and water temperature manipulations, are often economically unviable. Water disinfection strategies, e.g., using peracetic acid, target only free-swimming stages of the parasite (Rach et al. 2000), and bath treatments induce stress via fish handling and require large amounts of chemotherapeutics (Bodensteiner et al. 2000). These issues can be effectively mitigated using oral application of selected drugs, preferably including the mechanism of controlled release.

In this study, the measured concentrations of doxycycline in muscle with skin, whole fish homogenates and water provided insight into the distribution and persistence of the drug following oral administration of both types of medicated feed mixtures. Three days after treatment, detectable residues were present in all matrices, with the highest concentrations consistently found in whole-fish homogenates. The approximately threefold higher levels in whole fish than in muscle with skin suggest a non-uniform distribution of doxycycline within the body and preferential accumulation in internal organs. Comparable disparities between edible muscle tissue and whole-body residues have been documented for other tetracyclines, particularly oxytetracycline, in marine and freshwater fish (Malvisi et al. 1996), and are consistent with previous observations that residues tend to be substantially higher in liver, kidney, and other metabolically active organs than in muscle (VICH 2019; Corum et al. 2023). Differences in depletion kinetics across tissues have also been demonstrated in controlled studies evaluating temperature-dependent residue decline in serum, muscle, and visceral organs (Lee et al. 2022), further supporting the observed pattern.

Despite the 2.5-fold lower doxycycline content in the controlled-release diet (DOX2), residues measured in fish at three days post-treatment reached approximately one-third of those found in regular doxycycline (DOX1). When expressed as a proportion of the estimated total ingested dose, the proportion of retained drug was similar across formulations, indicating that the controlled-release matrix ensured efficient absorption. This relationship resembles patterns described in studies using coated or modified-release medicated feeds. For example, starch-based coating of oxytetracycline pellets reduced leaching into water but resulted in comparable muscle residue concentrations relative to uncoated feed at the same dose (Marques et al. 2023). Likewise, dose-dependent but predictable concentrations of florfenicol in plasma and tissues have been demonstrated in trout fed coated medicated diets across a range of dosing regimens (Mallik et al. 2023). Together, these findings support the interpretation that properly formulated controlled-release diets can maintain adequate tissue exposure while reducing the nominal drug dose.

By day 20 post-treatment, doxycycline residues in all fish samples had fallen below the quantification limit (<50 μg/kg), consistent with earlier studies showing rapid post-treatment decline of tetracyclines following 10-day oral exposure (Chen et al. 2004). Low but measurable concentrations persisted in water, decreasing by 56% (DOX1) and 77% (DOX2) between days 3 and 20. Since all feed was consumed immediately, waterborne residues most likely reflect excretion of unmetabolised doxycycline via faeces and urine. A slow decline in antibiotic residues in rearing water has been previously noted in both flow-through and static systems, with studies reporting that large proportions of administered antibiotics (often 70–80%) are excreted and can remain detectable for extended periods, depending on compound stability and environmental conditions (Watts et al. 2017).

Importantly, although DOX2 contained only 40% of the doxycycline present in DOX1, it produced comparable proportions of retained drug and, notably, resulted in superior protection against *Ich*. This alignment between reduced dosing, adequate residue levels, and strong antiparasitic efficacy is in accordance with reports showing that controlled-release or matrix-bound formulations can prolong drug exposure and enhance treatment outcomes while reducing the total antibiotic load (Neto et al. 2019; Tang et al. 2022). The present findings therefore indicate that the DOX2 formulation not only minimised tissue and environmental residues but also maintained biologically effective exposure levels sufficient to suppress trophont re-establishment and improve post-treatment survival.

Taken together, the residue data confirm that both medicated diets were effectively absorbed and subsequently depleted within the post-treatment period. The controlled-release diet achieved the dual benefit of reduced tissue and environmental loading while delivering improved antiparasitic efficacy, as confirmed by survival rates, underscoring its potential usefulness as an optimised medicated feed for salmonid aquaculture.

## References

[R1] Abdel-Hafez G, Lahnsteiner F, Mansour N. Possibilities to control Ichthyophthirius multifiliis infestation with medicated feed in rainbow trout (Oncorhynchus mykiss) and chub (Leuciscus cephalus). Parasitol Res. 2014 Mar;113(3):1119-26. 24419403 10.1007/s00436-013-3749-9

[R2] Bodensteiner LR, Sheehan RJ, Wills PS, Brandenburg AM, Lewis WM. Flowing water: An effective treatment for ichthyophthiriasis. J Aquat Anim Health. 2000 Sep;12(3):209-19.

[R3] Buchmann K. Control of parasitic diseases in aquaculture. Parasitology. 2022 Dec;149(14):1985-97. 35950444 10.1017/S0031182022001093 PMC10090776

[R4] Chen CY, Getchell RG, Wooster GA, Craigmill AL, Bowser PR. Oxytetracycline residues in four species of fish after 10-day oral dosing in feed. J Aquat Anim Health. 2004 Dec;16(4):208-19.

[R5] Corum O, Durna Corum D, Terzi E, Uney K. Pharmacokinetics, tissue residues, and withdrawal times of oxytetracycline in rainbow trout (Oncorhynchus mykiss) after single- and multiple-dose oral administration. Animals (Basel). 2023 Dec 14;13(24):3845. 38136882 10.3390/ani13243845 PMC10740422

[R6] European Commission. Commission implementing regulation (EU) 2015/151 of 30 January 2015 amending the annex to regulation (EU) No 37/2010 as regards the substance ‘doxycycline’. Off J Eur Union L. 2015 Jan 31;26(L 26):13-5.

[R7] European Commission. Commission regulation (EU) 2023/411 of 23 February 2023 amending regulation (EU) 2019/1871 as regards the application of reference points for action for nitrofurans and their metabolites. Off J Eur Union L. 2023 Feb 23;59(L 59):8-10.

[R8] FAO – Food and Agriculture Organization of the United Nations. Aquaculture development. 8. Recommendations for prudent and responsible use of veterinary medicines in aquaculture. FAO technical guidelines for responsible fisheries, No. 5, Suppl. 8. Rome (IT): FAO; 2019. 66 p.

[R9] Hough C. Regional review on status and trends in aquaculture development in Europe – 2020. FAO Fisheries and Aquaculture Circular, No. 1232/1. Rome (IT): FAO; 2022.

[R10] Ibrahim M, Ahmad F, Yaqub B, Ramzan A, Imran A, Afzaal M, Mirza SA, Mazhar I, Younus M, Akram Q, Taseer MSA, Ahmad A, Ahmad S. Current trends of antimicrobials used in food animals and aquaculture. In: Hashmi MZ, editor. Antibiotics and antimicrobial resistance genes in the environment. Amsterdam: Elsevier; 2020. p. 39-69.

[R11] Kim E, Park S, Park H, Choi J, Yoon HJ, Kim JH. Determination of anthelmintic and antiprotozoal drug residues in fish using liquid chromatography-tandem mass spectrometry. Molecules. 2021 Apr 28;26(9):2575. 33925124 10.3390/molecules26092575 PMC8125621

[R12] Lee JH, Kim GW, Kwon MG, Seo JS. Temperature-dependent tissue residue depletion and withdrawal time of orally administered tylosin tartrate in starry flounder, Platichthys stellatus. Aquaculture. 2022;561:738644.

[R13] Lunden T, Miettinen S, Lonnstrom LG, Lilius EM, Bylund G. Influence of oxytetracycline and oxolinic acid on the immune response of rainbow trout (Oncorhynchus mykiss). Fish Shellfish Immunol. 1998;8(3):217-30.

[R14] Mallik SK, Shahi N, Pathak R, Kala K, Patil PK, Singh B, Ravindran R, Krishna N, Pandey PK. Pharmacokinetics and biosafety evaluation of a veterinary drug florfenicol in rainbow trout, Oncorhynchus mykiss (Walbaum 1792) as a model cultivable fish species in temperate water. Front Pharmacol. 2023 Jan 23;14:1033170. 36755946 10.3389/fphar.2023.1033170 PMC9900004

[R15] Malvisi J, Della Rocca G, Anfossi P, Giorgetti G. Tissue distribution and residue depletion of oxytetracycline in sea bream (Sparus aurata) and sea bass (Dicentrarchus labrax) after oral administration. Aquaculture. 1996;147(3-4):159-68.

[R16] Marques AR, Cheng TH, Man KY, Cheng KP, Cheung KB, St-Hilaire S. Evaluation of oxytetracycline leaching from pregelatinized starch-coated medicated fish feed. J Fish Dis. 2023 Nov;46(11):1183-92. 37477182 10.1111/jfd.13838

[R17] Neto PF, da Silva AFB, Moro EB, Pilarski F, de Freitas O, Mooney MH, Paschoal JAR. Emamectin benzoate in tilapia: Alternative method for drug incorporation into feed and associated residual depletion study. Food Res Int. 2019 May;119:524-9. 30884685 10.1016/j.foodres.2019.01.033

[R18] Okada M, Guo P, Nalder SA, Sigala PA. Doxycycline has distinct apicoplast-specific mechanisms of antimalarial activity. Elife. 2020 Nov 2;9:e60246. 33135634 10.7554/eLife.60246 PMC7669263

[R19] Rach JJ, Gaikowski MP, Ramsay RT. Efficacy of hydrogen peroxide to control parasitic infestations on hatchery-reared fish. J Aquat Anim Health. 2000;12(4):267-73.

[R20] Sun HY, Noe J, Barber J, Coyne RS, Cassidy-Hanley D, Clark TG, Findly RC, Dickerson HW. Endosymbiotic bacteria in the parasitic ciliate Ichthyophthirius multifiliis. Appl Environ Microbiol. 2009 Dec;75(23):7445-52. 19820157 10.1128/AEM.00850-09 PMC2786411

[R21] Tang EKY, Partridge GJ, Woolley LD, Pilmer L, Lim LY. Effects of formulation on the palatability and efficacy of in-feed praziquantel medications for marine finfish aquaculture. Mar Drugs. 2022 May 13;20(5):323. 35621974 10.3390/md20050323 PMC9144810

[R22] VICH – Veterinary International Conference on Harmonisation. Studies to evaluate the metabolism and residue kinetics of veterinary drugs in food-producing species: Marker residue depletion studies to establish product withdrawal periods in aquatic species (VICH GL57). Document No. EMA/CVMP/VICH/517152/2013. London: European Medicines Agency (EMA); 2019.

[R23] Watts JEM, Schreier HJ, Lanska L, Hale MS. The rising tide of antimicrobial resistance in aquaculture: Sources, sinks and solutions. Mar Drugs. 2017 Jun 1;15(6):158. 28587172 10.3390/md15060158 PMC5484108

